# Direct and indirect costs and cost-driving factors of Tuberous sclerosis complex in children, adolescents, and caregivers: a multicenter cohort study

**DOI:** 10.1186/s13023-021-01899-x

**Published:** 2021-06-21

**Authors:** Janina Grau, Johann Philipp Zöllner, Susanne Schubert-Bast, Gerhard Kurlemann, Christoph Hertzberg, Adelheid Wiemer-Kruel, Thomas Bast, Astrid Bertsche, Ulrich Bettendorf, Barbara Fiedler, Andreas Hahn, Hans Hartmann, Frauke Hornemann, Ilka Immisch, Julia Jacobs, Matthias Kieslich, Karl Martin Klein, Kerstin A. Klotz, Gerhard Kluger, Markus Knuf, Thomas Mayer, Klaus Marquard, Sascha Meyer, Hiltrud Muhle, Karen Müller-Schlüter, Anna H. Noda, Susanne Ruf, Matthias Sauter, Jan-Ulrich Schlump, Steffen Syrbe, Charlotte Thiels, Regina Trollmann, Bernd Wilken, Laurent M. Willems, Felix Rosenow, Adam Strzelczyk

**Affiliations:** 1 Epilepsy Center Frankfurt Rhine-Main and Department of Neurology, Goethe-University Frankfurt, Schleusenweg 2-16 (Haus 95), 60528 Frankfurt am Main, Germany; 2 Center for Personalized Translational Epilepsy Research (CePTER), Goethe-University Frankfurt, Frankfurt am Main, Germany; 3 Department of Neuropediatrics, Goethe-University Frankfurt, Frankfurt am Main, Germany; 4 St. Bonifatius Hospital, Lingen, Germany; 5 Department of Neuropediatrics, Vivantes Klinikum Neukölln, Berlin, Germany; 6 Epilepsy Center Kork, Clinic for Children and Adolescents, Kehl-Kork, Germany; 7 Department of Neuropediatrics, University Hospital for Children and Adolescents, Rostock, Germany; 8 Neuropediatric Practice, Hirschaid, Germany; 9 Department of General Pediatrics, Division of Neuropediatrics, University Hospital Münster, Münster, Germany; 10 Department of Neuropediatrics, Justus-Liebig-University Gießen, Gießen, Germany; 11 Department of Neuropediatrics, Clinic for Pediatric Kidney, Liver and Metabolic Diseases, Hannover Medical School, Hannover, Germany; 12 Department of Neuropediatrics, Leipzig University Hospital for Children and Adolescents, Leipzig, Germany; 13 Epilepsy Center Hessen and Department of Neurology, Philipps-University Marburg, Marburg (Lahn), Germany; 14 Department of Neuropediatrics and Muscle Disorders, Center for Pediatrics, Medical Center, Faculty of Medicine, University of Freiburg, Freiburg i.Br., Germany; 15 Department of Pediatrics and Clinical Neurosciences, Cumming School of Medicine, University of Calgary, Calgary, AB Canada; 16 Departments of Clinical Neurosciences, Medical Genetics and Community Health Sciences, Hotchkiss Brain Institute & Alberta Children’s Hospital Research Institute, Cumming School of Medicine, University of Calgary, Calgary, AB Canada; 17 Berta-Ottenstein-Programme, Faculty of Medicine, University of Freiburg, Freiburg i.Br., Germany; 18 Clinic for Neuropediatrics and Neurorehabilitation, Epilepsy Center for Children and Adolescents, Schön Clinic Vogtareuth, Vogtareuth, Germany; 19 Research Institute, Rehabilitation, Transition and Palliation, PMU Salzburg, Salzburg, Austria; 20 Department of Pediatrics, Klinikum Worms, Worms, Germany; 21 Department of Pediatrics, University Medicine Mainz, Mainz, Germany; 22 Epilepsy Center Kleinwachau, Dresden-Radeberg, Germany; 23 Department of Pediatric Neurology, Psychosomatics and Pain Management, Klinikum Stuttgart, Stuttgart, Germany; 24 Department of Neuropediatrics, Children’s Hospital at University Medical Center Homburg, Homburg, Germany; 25 Department of Neuropediatrics, Christian-Albrechts-University Kiel & University Hospital Schleswig-Holstein, Campus Kiel, Kiel, Germany; 26 Epilepsy Center for Children, Brandenburg Medical School, University Hospital Neuruppin, Neuruppin, Germany; 27 Department of Neuropediatrics, University Hospital Tübingen, Tübingen, Germany; 28 Klinikum Kempten, Klinikverbund Allgäu, Kempten (Allgäu), Germany; 29 Department of Neuropediatrics, University of Witten/Herdecke, Herdecke, Germany; 30 Division of Pediatric Epileptology, Centre for Paediatrics and Adolescent Medicine, University Hospital Heidelberg, Heidelberg, Germany; 31 Department of Neuropediatrics and Socialpediatrics, University Hospital of Ruhr University Bochum, Bochum, Germany; 32 Department of Neuropediatrics, Friedrich-Alexander University of Erlangen-Nürnberg, Erlangen, Germany; 33 Department of Neuropediatrics, Klinikum Kassel, Kassel, Germany

**Keywords:** mTOR inhibitor, Everolimus, Seizure, Rhabdomyoma, Epilepsy, Anti-seizure medication

## Abstract

**Background:**

Tuberous sclerosis complex (TSC), a multisystem genetic disorder, affects many organs and systems, characterized by benign growths. This German multicenter study estimated the disease-specific costs and cost-driving factors associated with various organ manifestations in TSC patients.

**Methods:**

A validated, three-month, retrospective questionnaire was administered to assess the sociodemographic and clinical characteristics, organ manifestations, direct, indirect, out-of-pocket, and nursing care-level costs, completed by caregivers of patients with TSC throughout Germany.

**Results:**

The caregivers of 184 patients (mean age 9.8 ± 5.3 years, range 0.7–21.8 years) submitted questionnaires. The reported TSC disease manifestations included epilepsy (92%), skin disorders (86%), structural brain disorders (83%), heart and circulatory system disorders (67%), kidney and urinary tract disorders (53%), and psychiatric disorders (51%). Genetic variations in *TSC2* were reported in 46% of patients, whereas 14% were reported in *TSC1*. Mean total direct health care costs were EUR 4949 [95% confidence interval (95% CI) EUR 4088–5863, median EUR 2062] per patient over three months. Medication costs represented the largest direct cost category (54% of total direct costs, mean EUR 2658), with mechanistic target of rapamycin (mTOR) inhibitors representing the largest share (47%, EUR 2309). The cost of anti-seizure drugs (ASDs) accounted for a mean of only EUR 260 (5%). Inpatient costs (21%, EUR 1027) and ancillary therapy costs (8%, EUR 407) were also important direct cost components. The mean nursing care-level costs were EUR 1163 (95% CI EUR 1027–1314, median EUR 1635) over three months. Total indirect costs totaled a mean of EUR 2813 (95% CI EUR 2221–3394, median EUR 215) for mothers and EUR 372 (95% CI EUR 193–586, median EUR 0) for fathers. Multiple regression analyses revealed polytherapy with two or more ASDs and the use of mTOR inhibitors as independent cost-driving factors of total direct costs. Disability and psychiatric disease were independent cost-driving factors for total indirect costs as well as for nursing care-level costs.

**Conclusions:**

This study revealed substantial direct (including medication), nursing care-level, and indirect costs associated with TSC over three months, highlighting the spectrum of organ manifestations and their treatment needs in the German healthcare setting.

*Trial registration*: DRKS, DRKS00016045. Registered 01 March 2019, http://www.drks.de/DRKS00016045.

**Supplementary Information:**

The online version contains supplementary material available at 10.1186/s13023-021-01899-x.

## Key Point

- First study to measure both direct and indirect costs of children with TSC and their caregivers.
- Mean total direct costs were estimated to be EUR 4949 for three months.
- Medication, especially mTOR inhibitors, and hospitalization are major direct cost components.
- Total indirect costs were higher for mothers (mean EUR 2813 for three months) than fathers (EUR 372).
- Total costs are driven by the number of TSC manifestations and affected organ systems.

## Background

Tuberous sclerosis complex (TSC) is a rare, multisystem, genetic disorder that affects up to 1 in 5,000 individuals worldwide. Until recently, the prevalence of TSC was underestimated due to incomplete penetrance and considerable inter-individual phenotypic variability among those affected by TSC [1–6]. The clinical manifestation of TSC undergoes a typical pattern of changes during life, in which multiple organs and systems are affected, leading to the development of typically benign tumors that present preferentially in the skin, brain, and kidneys. Most individuals with TSC suffer from structural epilepsy due to the presence of cortical tubers or other cortical malformations. The clinical picture may differ considerably among patients and range from very limited manifestations to severe impairments, requiring nursing assistance [3, 7].

TSC is caused by a loss of function mutation in one of two tumor suppressor genes, *TSC1* or *TSC2* (ratio 1:3.4, as reported in [8]), and is inherited in an autosomal-dominant fashion; however, the majority of cases appear to be caused by de novo mutations. Genetic mosaicism and deep intronic mutations may contribute to disease development in the 15% of cases associated with no definitive hereditary mutation, despite a definite clinical diagnosis of TSC [8]. Loss of function mutations in *TSC1* or *TSC2* result in deregulated expression patterns for components of the mechanistic target of rapamycin (mTOR) pathway, resulting in the abnormal production of end products, which ultimately promotes tumorigenesis [9]. Treatment with mTOR inhibitors addresses the underlying cause and might prevent epileptogenesis and late organ manifestations [10].

The burden of illness in TSC is considerable and directly associated with the complex and multifaceted disease manifestations [11–15]. Several studies examining the cost-of-illness (COI) and their predictors in TSC have been published over the last two decades; however, only a few have addressed both direct costs and cost-driving factors, and the indirect costs experienced by the caregivers of children with TSC have not yet been addressed [3]. Furthermore, the majority of available studies evaluated patients before the availability of mTOR inhibitors, such as everolimus, which has been approved for the treatment of various organ manifestations in TSC [3, 16].

Thus, the present study aimed to provide a comprehensive analysis of the direct and indirect costs and potential cost-driving factors among a large, multicenter cohort of children, adolescents, and their caregivers in Germany.

## Methods

### Patients and recruitment

This study was designed as a cross-sectional, multicenter survey, which enrolled patients with TSC and their caregivers through the German TSC patient advocacy group (Tuberöse Sklerose Deutschland e.V., Wiesbaden, Germany) and from centers throughout Germany (Berlin, Bochum, Dresden [Radeberg], Erlangen, Frankfurt, Freiburg, Giessen, Hannover, Herdecke, Heidelberg, Hirschaid, Homburg, Kassel, Kiel, Kork, Leipzig, Lingen, Marburg, Münster, Neuruppin, Oberhausen, Rostock, Stuttgart, Tübingen, Vogtareuth, and Wiesbaden).

### Survey methods

After receiving written informed consent from the patients’ parents or legal guardians, all patients with TSC and their caregivers were deemed eligible. The diagnostic criteria for TSC were based on the latest recommendations, which were established by the 2012 International TSC Consensus Conference [17]. Seven primary manifestation categories associated with TSC were identified, including epilepsy, structural brain disorders, psychiatric disorders, heart/circulatory system disorders, kidney and urinary tract disorders, skin disorders, respiratory system disorders, and other manifestations [11]. The seizure and epilepsy syndrome classifications were adapted according to the latest definitions established by the International League Against Epilepsy (ILAE) [18, 19]. This study received ethics approval and was registered with the German Clinical Trials Register (DRKS00016045; Universal Trial Number: U1111-1229-4714). The STROBE guidelines (Strengthening The Reporting of Observational Studies in Epidemiology) were closely followed [20].

The caregivers of patients with TSC were asked to complete a retrospective questionnaire referencing the previous three months. The questionnaire, which was validated in previous studies [21–23] and adapted for use in patients with TSC, comprised 36 questions regarding disease characteristics (e.g., genetics, affected organ systems, seizure occurrence, medication use, and additional symptoms), healthcare resource use (e.g., healthcare visits, accidents, and emergency care), and social conditions. Paper questionnaires were mailed to caregivers in Germany between February and July 2019.

### Costing methods

The aim of this study was to calculate the genuine costs associated directly with TSC, rather than the costs associated with conditions other than TSC. Therefore, caregivers were asked, in detail, whether the medications, services, and other medical resources used were specifically associated with TSC organ manifestations. Costs were evaluated through a bottom-up approach from the perspective of the statutory health insurer (“Gesetzliche Krankenversicherung” [GKV]), the patients and society as a whole. The cost categories that were included in this analysis included direct health service costs covered by the statutory health insurance and as patients’ and caregivers’ out-of-pocket (OOP) expenses, nursing care-level costs covered by the statutory care insurance, and further informal care not covered by any statutory insurance, and indirect costs. Costs were evaluated according to the German recommendations for performing economic evaluations related to healthcare [24].

#### Direct healthcare costs

Direct health service costs, which included inpatient stays, outpatient visits, medicines [anti-seizure drugs (ASDs), mTOR inhibitors, other prescription drugs, over-the-counter drugs, and emergency medications], medical aids, healthcare professional visits, emergency transportation, diagnostic studies, specific diets, patients’ co-payments, rehabilitation costs, private transport costs, and co-payments for therapies, were drawn from the literature and standard reference sources for Germany and were estimated as previously described [21, 25]. Drug costs were based on the Drug Prescription Report of 2019 (“Arzneiverordnungs-Report”) [26], which is an index of available medicines and their average prices in Germany. The costs of inpatient and outpatient care, specialist care, therapies, and diagnostic studies were standardized, according to Bock et al. [27] and physician fee scales (Einheitlicher Bewertungsmaßstab) [28]. All costs were inflated to 2019 levels using the consumer price index for Germany and expressed in both annual and 3-month terms based on the 2019 value of the Euro.

#### Out-of-pocket (OOP) expenses

OOP expenses (co-payments) were reported by respondents. For situations in which supply-side utilization estimates were not available (care and supervision, healing agents, and diets), and for those expenditures outside of formal healthcare settings (alternative and occupational therapies and equipment costs), the reported costs were listed as OOP expenses and added to total direct healthcare costs. For instances in which supply-side cost estimates were calculated according to resource utilization (ancillary treatments, medical aids, healthcare professionals, and emergency transportation), OOP expenses were considered to be accounted for, and were not added to total direct costs to prevent double accounting.

#### Nursing care-level costs and grade of disability

In Germany, care insurance payments are determined by the patient care grade, which ranges from Level 1 to 5 on the Pflegegrade scale (which categorizes the need for care), and determines the basis for the care allowances that are paid by the German statutory care insurance “Pflegeversicherung” [29]. Care grade levels depend on the time needed per day in minutes for care in daily life. Average care grade allowances were calculated based on the assumption that nursing services were being provided by family members. Nursing care-level costs in this study may be interpreted as a proxy for overall informal care costs. Additional informal care costs that were reported and paid by the respondents were considered separately.

The “grade of disability” is assigned in the German social system to people with disabilities who are entitled to certain monetary and social compensations for their disadvantages. The grade of disability quantifies the type and severity of a disability, upon which these compensations depend. Grade of disability is classified by an independent medical professional (“Versorgungsamt”) and if applicable can vary between 20 and 100, in steps of ten.

#### Indirect costs

Productivity losses associated with the need to care for TSC patients were calculated for caregivers of working age (i.e., below the age of 67 years) using the human capital approach for days off, quitting work, and reductions in working hours. Productivity losses due to TSC among adolescent patients of working age (i.e., > 16 years of age) were considered in terms of days off for working adolescents and the inability to work among those who were completely unable to work or attend school. A mean gross wage of EUR 44,964 in 2019 [30] was assumed for calculating the productivity costs for caregivers who quit their jobs. To account for the costs of days taken off work to care for a child with TSC, annual gross wages were calculated to represent EUR 215 per calendar day, and this daily income was multiplied by the number of days off [22].

#### Grouping of questionnaire items

Some questionnaire items were collated into groups when presenting the results, as follows: ‘ancillary costs’: physiotherapy, speech therapy, occupational therapy, acupuncture, hippotherapy, and other ancillary costs; ‘healthcare professionals’: neurologists, general practitioners, orthopedic surgeons, child psychiatrists, alternative medicine practitioners, homeopathy, dietitians, and other specialists; and ‘diagnostic studies’: electroencephalography (EEG), blood tests, magnetic resonance imaging (MRI) or computed tomography (CT) scans, X-rays, and other diagnostic studies.

### Data availability statement

The reported data and the questionnaire are available to qualified researchers upon reasonable request.

### Statistical analysis

Statistical analysis was performed using IBM SPSS Statistics, version 26 (IBM Corp., Armonk, NY, USA). The variables of interest were summarized using the mean, median, and standard deviation (SD). For cost data, the 95% confidence interval (95% CI) was calculated using the bootstrap-corrected and accelerated method, considering the fact that most cost variables are highly skewed [31]. Comparisons between groups were performed using adequate parametric and nonparametric tests. Significance was assumed at *p* < 0.05. The relationship between patient characteristics and TSC-related costs was investigated using multivariate regression. Total direct, total indirect, and nursing care-level costs were regressed on a set of clinical variables that were selected following univariate analyses and according to evidence from previous cost-of-illness studies for TSC [11, 32, 33]. All variables were tested for interactions and collinearity. Standard multiple regression analysis using the bootstrapping technique was performed to identify independent predictors of costs, and a Bonferroni correction was applied for multiple testing.

## Results

### Demographic and clinical characteristics

One hundred and eighty-four caregivers of children and adolescents with TSC completed the questionnaire. The mean patient age was 9.8 years (SD 5.3 years, median 9.8 years; range 0.7–21.8 years), and 48.4% (n = 89) of patients were females. TSC was diagnosed at a mean age of 1.3 years (SD 2.1 years, median 0.5 years; range 0–12.1 years), and the first symptoms of TSC were observed at a mean age of 0.8 years (SD 1.3 years, median 0.4 years; range 0–7.7 years). In 34 children (18.5%), a diagnosis of TSC was suspected before birth based on the findings of ultrasound examinations. In 25 patients (13.6%), variations were identified in *TSC1,* whereas in 85 patients (46.2%), variations were observed in *TSC2* (*TSC1:TSC2* ratio 1:3.4), and five patients (2.7%) suffered from a polycystic kidney disease with tuberous sclerosis (PKDTS), which is a contiguous gene deletion syndrome. Most children lived with both parents at home (n = 151, 82.1%). Forty percent (n = 74) of patients attended a school with a special focus on learning, and mental and physical development, whereas the majority of small children attended kindergarten (n = 49, 26.6%). Among the respondents, mothers had a mean age of 41 years, and fathers were older, on average, with a mean age of 43 years. The occupational situations of parents revealed that 70% of mothers (n = 128) and 91% of fathers (n = 167) were employed. Additional sociodemographic and clinical characteristics and affected family members are presented in Table 1. The majority of patients were affected by a range of TSC organ manifestations, including 169 (91.8%) patients diagnosed with epilepsy, 158 (85.9%) showing skin manifestations, 153 (83.2%) presenting with various structural brain disorders, and 123 (66.8%) reporting heart and circulatory system disorders. Additional details can be found in Table 2.

**Table 1:** Sociodemographic and clinical characteristics (n = 184)

	All patients n = 184
Age in years^1^	9.8 ± 5.3
	range
	0.7–21.8
Sex	% (n)
Male	51.6 (95)
Female	48.4 (89)
Age at first symptoms due to TSC^1^	0.8 ± 1.3
	range
	0–7.7
Age at TSC diagnosis in years^1^	1.3 ± 2.1
	range
	0–12.1
TSC diagnosis before birth by ultrasound	% (n)
No	81.5 (150)
Yes	18.5 (34)
Genetics	% (n)
TSC1-gene	13.6 (25)
TSC2-gene	46.2 (85)
TSC2/PKD1 contiguous-gene	2.7 (5)
No genetic test	15.2 (28)
No genetic mutation	4.9 (9)
Unknown	17.3 (32)
Affected family members by TSC	% (n)
No	85.3 (157)
Yes	14.7 (27)
* Mother affected (23.9 years)*^*2*^	*7.6 (14)*
* Father affected (33.6 years)*^*2*^	*6.5 (12)*
* Sibling affected (8.8 years)*^*2*^	*4.9 (9)*
* Grandparents affected*	*2.2 (4)*
Parents' age in years^1^	
Mother	40.8 ± 7.0
Father	43.4 ± 7.4
Living conditions	% (n)
With mother and father	82.1 (151)
Only with mother	13.0 (24)
Only with father	0.5 (1)
Other	4.4 (8)
Childcare and School	% (n)
School for children with special needs^3^	40.2 (74)
Kindergarten	26.6 (49)
Primary mainstream school	10.9 (20)
Secondary mainstream school	8.2 (15)
Only at home	6.0 (11)
Day care	0.5 (1)
Employed	0.5 (1)
Unknown/other	7.1 (13)

^1^Mean ± standard deviation

^2^Mean age at TSC diagnosis of affected family members

^3^Learning, mental and physical development

**Table 2:** Organ manifestations in TSC patients^1^ (n = 184)

	%	n
Epilepsy	91.8	169
Recurrent seizures	47.8	88
Seizure free > 1 year or no seizures	52.2	96
Structural brain disorders	83.2	153
SEGA^2^	33.2	61
Cortical tubers	76.1	140
Hydrocephalus	3.3	6
Psychiatric disorders	51.1	94
Heart and circulatory system	66.8	123
Rhabdomyomas	61.4	113
Hypertension	6.0	11
Aneurysm Aorta	1.1	2
Arrhythmia	7.6	14
Kidney and urinary tract	53.3	98
Chronic kidney dysfunction	1.6	3
Angiomyolipomas	45.7	84
Cysts	29.9	55
Skin manifestations	85.9	158
Hypomelanotic macules	74.5	137
Angiofibromas	60.3	111
Shagreen patches	39.1	72
Forehead plaque	3.3	6
Ungal/periungal fibromas	1.1	2
Other disorders	29.9	55
Iris or retinal hamartomas/astrocytomas	19.0	35
Angiomyolipomas in other organ systems^3^	3.3	6
Cysts in other organ systems^3^	2.7	5

^1^Respiratory system manifestations were not reported

^2^Subependymal giant cell astrocytoma

^3^Hormone system, Thyroid, Gastrointestinal, Liver, Spleen, Pancreas

### Direct costs

**Fig. 1:**
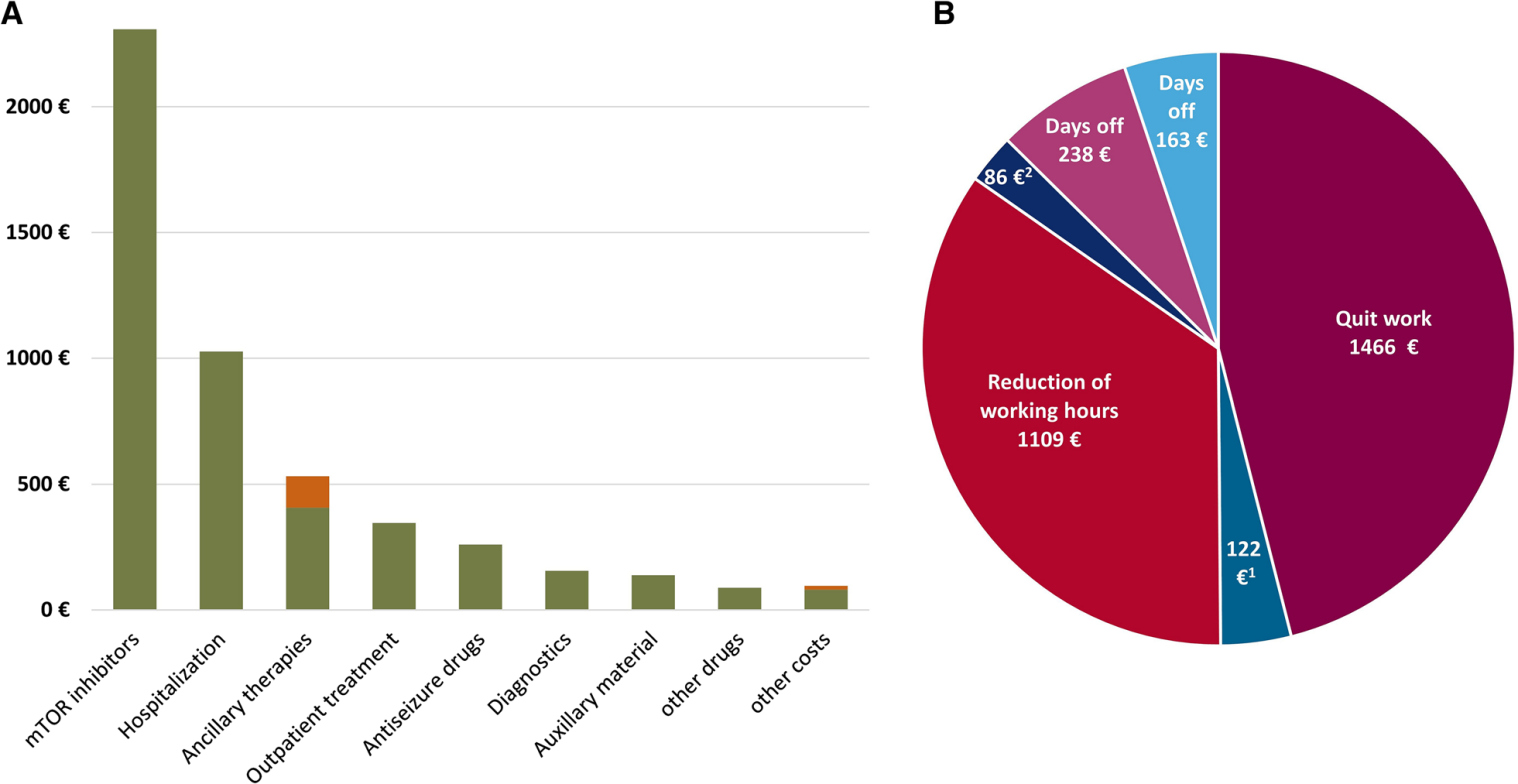
Breakdown of total direct costs (**A**), with co-payments in orange, per patient over 3 months and total indirect costs for caregivers over 3 months (**B**). Maternal costs are in red and paternal costs are in blue. ^1^quit work; ^2^reduction of working hours

The mean total direct costs were calculated at EUR 4949 (95% CI: EUR 4088–5863, median: EUR 2062) per study participant for the reported 3-month study period, and details are presented in Table 3 and Fig. 1A. Direct medical costs were primarily associated with the costs of drug treatment (53.7% of total direct costs, mean: EUR 2658 per 3 months, 95% CI: EUR 2060–3297, median: EUR 321) and hospitalization (20.8% of total direct costs, mean: EUR 1027, 95% CI EUR 579–1503; median EUR 0).

**Table 3:** Direct costs for a 3-month period for the total patient group (n = 184; in 2019 Euro)

Cost components	Mean costs	SD^1^	Minimum	Median	Maximum	95% CI	% of total direct costs	Estimated annual direct costs^2^
Total direct costs	4949	6079	0	2062	29,231	4088; 5863	100	19,796
Medication (n = 168)	2658	4557	0	321	21,546	2060; 3297	53.7	10,632
mTOR inhibitors* (n = *49)*	*2309*	*4435*	*0*	*0*	*20,054*	*1715; 2928*	*46.6*	*9236*
* Antiseizure drugs (n* = *154)*	*260*	*378*	*0*	*159*	*3027*	*213; 312*	*5.3*	*1040*
* Other prescription drugs (n* = *62)*	*72*	*352*	*0*	*0*	*3569*	*33; 122*	*1.5*	*288*
* OTC drugs and supplements (n* = *30)*	*11*	*44*	*0*	*0*	*349*	*6; 17*	*0.2*	*44*
* Emergency medication (n* = *23)*	*5*	*24*	*0*	*0*	*266*	*2; 9*	*0.1*	*20*
Hospitalization (n = 41)	1027	3467	0	0	26,802	579; 1503	20.8	4108
Ancillary therapies (n = 116)	407	470	0	212	1951	345; 472	8.2	1628
Outpatient treatment (n = 169)	346	357	0	255	2250	298; 396	7.0	1384
Diagnostics (n = 153)	156	194	0	101	1370	130; 186	3.2	624
Auxillary material (n = 19)	138	738	0	0	8130	52; 241	2.8	552
Rehabilitation (n = 2)	27	287	0	0	3671	0; 67	0.6	108
Emergency service use (n = 5)	23	145	0	0	1200	7; 46	0.5	92
Specific diets (n = 11)	22	122	0	0	1100	8; 41	0.4	88
Transport costs (n = 51)	9	34	0	0	374	5; 14	0.2	36
Co-payments for therapies (n = 64)	125	297	0	0	2020	87; 167	2.5	500
Other co-payments (n = 22)	15	54	0	0	400	8; 23	0.3	60

^1^Standard deviation

^2^Estimation based on the mean costs in three months multiplied by four

95% CI = 95% Confidence interval using the bootstrap bias corrected and accelerated method

^*^Everolimus n = 46, Sirolimus n = 3, OTC = over-the-counter

Costs for mTOR inhibitors (everolimus, n = 46 and sirolimus, n = 3) were the primary direct cost components with a mean of EUR 2309 (46.6% of total direct costs, 95% CI: EUR 1715–2928, median: EUR 0), which was greater than the costs associated with ASDs (synonymous to anticonvulsants or antiepileptic drugs) which were associated with a mean cost of EUR 260 (5.3% of total direct costs, 95% CI: EUR 213–312, median: EUR 159). The patients used a mean number of 1.8 ASDs (SD 0.8, median 2, range 0–4). The five most frequently prescribed ASDs included oxcarbazepine (n = 49; 26.6%), vigabatrin (n = 48, 26.1%), lamotrigine (n = 47; 25.5%), valproate (n = 46; 25%), and levetiracetam (n = 25; 13.6%). ASD monotherapy was prescribed to 32.6% (n = 60) of all participants and was associated with lower costs than ASD polytherapy, comprising two or more ASDs (each *p* < 0.001). The detailed costs and daily dosages of the various ASDs are listed in Table 4.

**Table 4:** Prescription patterns and costs of anti-seizure drugs (ASDs) for a 3-month period (in 2019 Euro)

Medication costs	n	Mean costs per 3 months	SD^1^	Minimum	Median	Maximum	95% CI	*p* value^2^
All patients	184	€ 260	378	€ 0	€ 159	€ 3027	€ 213; 312	
No ASDs (16.3%)	30	0						
Monotherapy (32.6%)	60	€ 158	211	€ 7	€ 102	€ 1369	€ 117; 208	< 0.001^3^
2 ASDs (37.0%)	68	€ 283	191	€ 27	€ 241	€ 810	€ 242; 328	< 0.001^4^
≥ 3 ASDs (14.1%)	26	€ 736	708	€ 167	€ 519	€ 3027	€ 503; 994	< 0.004^5^

Prescribed medication	n	Mean daily dose	SD^1^	Minimum	Median	Maximum	Mean costs per 3 months	SD^1^
Oxcarbazepine (26.6%)	49	1006 mg	532 mg	150 mg	900 mg	2700 mg	€ 140	74
Vigabatrine (26.1%)	48	1480 mg	754 mg	500 mg	1250 mg	3500 mg	€ 267	136
Lamotrigine (25.5%)	47	213 mg	163 mg	6 mg	200 mg	700 mg	€ 53	41
Valproate (25.0%)	46	850 mg	373 mg	150 mg	840 mg	1650 mg	€ 39	17
Levetiracetam (13.6%)	25	1574 mg	1287 mg	500 mg	900 mg	5250 mg	€ 105	86
Lacosamide (6.5%)	12	284 mg	91 mg	120 mg	275 mg	400 mg	€ 619	198
Clobazame (4.9%)	9	29 mg	50 mg	3 mg	13 mg	150 mg	€ 78	136
Topiramate (3.3%)	6	194 mg	132 mg	38 mg	150 mg	400 mg	€ 128	87
Brivaracetam (2.7%)	5	195 mg	67 mg	125 mg	200 mg	300 mg	€ 450	155
Ethosuximide (2.7%)	5	440 mg	134 mg	300 mg	500 mg	600 mg	€ 79	24
Sulthiame (2.2%)	4	106 mg	69 mg	25 mg	113 mg	175 mg	€ 64	42
Carbamazepine (2.2%)	4	963 mg	256 mg	600 mg	1025 mg	1200 mg	€ 47	13
Phenytoin (1.6%)	3	250 mg	87 mg	200 mg	200 mg	350 mg	€ 20	7
Rufinamid (1.6%)	3	1333 mg	945 mg	600 mg	1000 mg	2400 mg	€ 799	566
Zonisamide (1.6%)	3	147 mg	50 mg	100 mg	140 mg	200 mg	€ 243	83
Other ASDs* (4.9%)	9							

^1^Standard deviation, 95% CI = 95% Confidence interval using the bootstrap bias corrected and accelerated method

^2^Mann–Whitney-U-test; ^3^Monotherapy vs. ≥ 3 ASDs^, 4^Monotherapy vs. 2 ASDs^, 5^2 ASDs vs ≥ 3 ASDs

^*^(Cannabidiol n = 2, Eslicarbazepine acetate n = 1, Felbamate n = 1, Perampanel n = 2, Phenobarbital n = 2, Potassium bromide n = 1)

In total, 41 (22.3%) of the included children and adolescents were admitted at least once to a hospital because of TSC during the 3-month study period. Overall, 49 admissions were reported, with a mean length of stay of 6.04 days (SD 7.5 days; median 3 days, range 1–42 days). Seizures were the reported reason for 23 admissions, whereas 22 admissions were due to diagnostics, and four additional admissions were associated with other TSC-related causes.

Ancillary treatments, such as occupational therapy, physiotherapy, and speech therapy were prescribed to 116 participants (63%), and with costs as high as EUR 407 per 3 months, representing 8.2% of total direct costs (95% CI: EUR 345–472, median: EUR 212). In addition, EUR 125 in the 3-month study period were directly paid by the families for therapies.

### Care needs and nursing care-level costs

Sixty-five percent (n = 120) of patients were categorized as meeting the care levels defined by the “Pflegebedürftigkeits scale” (2.7% Level I [‘low impairment of independence’]; 12% Level II [‘significant need for care’]; 20.7% Level III [‘heavy need for care’]; 18.5% Level IV [‘most difficult to care for’]; and 11.4% Level V [‘most difficult to care for and special demands of nursing care’]). Approximately 3% (n = 5) of patients did not meet the Level I–V criteria but were reported as being in need of care according to their caregivers. Only 32% of patients were reported as not in need of care. The costs for nursing care were calculated as EUR 1163 (95% CI: EUR 1027–1314, median: EUR 1635) per 3-month period and EUR 4652 annually, assuming that care is provided by family members. Parents reported that they incurred further costs for informal care, with a mean of EUR 20.8, and supervision, with a mean of EUR 27.6 per 3-month period. In total, 123 patients (66.8%) had a severely disabled pass between 70% and 100% (maximum = 100%). No disability or disability ≤ 60% were identified in 33% (n = 61) of patients.

### Indirect (productivity) costs

All parents were of working age, and lost work time was recorded separately for mothers and fathers. In total, 111 parents reported that they had changed their working situation or remained out of work due to TSC in their child. Total indirect costs were calculated at a mean of EUR 3184 (95% CI: EUR 2533–3811, median: EUR 645) over three months, or EUR 12,736 annually. Twenty-four mothers (13.0%, compared with 1.1% of fathers) reported that they quit working, 49 mothers reduced their working hours (26.6%, compared with 3.8% of fathers), and 41 mothers missed days of work during the last three months due to TSC (22.3%, the same number of 41 [22.3%] who missed days of work applied to fathers). Mean productivity costs over three months were estimated at EUR 1466, associated with mothers quitting work (EUR 122 for fathers); EUR 1109, associated with mothers’ reduced working hours (EUR 86 for fathers); and EUR 238 associated with mothers’ lost workdays (EUR 163 for fathers). The total mean maternal indirect costs totaled EUR 2813 (95% CI: EUR 2221–3394, median: EUR 215,) over three months, or EUR 11,252 annually, whereas the total indirect costs for fathers were calculated at EUR 372 (95% CI: EUR 193–586, median: EUR 0) over three months, or EUR 1488 annually. These details are provided in Table 5 and Fig. 1B.

**Table 5:** Indirect costs to caregivers for a 3-month period (in 2019 Euro)

Indirect costs components	n^1^	Mean costs	SD^2^	Minimum	Median	Maximum	95% CI	Estimated annual costs^3^
Maternal indirect costs	96	2813	3950	0	215	11,241	2221; 3394	11,252
Quit work	24	1466	3796	0	0	11,241	916; 2016	5864
Reduction of working hours	49	1109	2136	0	0	8774	808; 1438	4436
Days off due to TSC	41	238	628	0	0	4300	153; 332	952
Paternal indirect costs	48	372	1345	0	0	11,241	193; 586	1488
Quit work	2	122	1169	0	0	11,241	0; 306	488
Reduction of working hours	7	86	531	0	0	5621	22; 169	344
Days off due to TSC	41	163	452	0	0	3440	101; 233	652
Total parents	111	3184	4326	0	645	22,482	2533; 3811	12,736

95% CI = 95% Confidence interval using the bootstrap bias corrected and accelerated method

^1^Parents of working age

^2^Standard deviation

^3^Estimation based on the mean costs in three months multiplied by four

Twenty-five (13.6%) of the adolescents included in this study were older than 16 years of age. Five of them were working, two were unable to work or attend school due to TSC, and 18 were in school, at university, or in vocational training. The mean indirect costs were calculated at EUR 1002 (95% CI: EUR 103–2005, median: EUR 0) per 3 months, which were due to 12 days off work due to TSC in one adolescent and the two individuals who were unable to work or attend school.

### Cost drivers of direct, indirect, and nursing care-level costs

To identify potential cost-driving factors, we performed univariate analyses of total direct, total indirect, and nursing care-level costs, and a number of demographic and clinical patient characteristics. The indirect costs of mothers and fathers were considered together. Polytherapy with two or more ASDs, the use of mTOR inhibitors, TSC manifestations, such as epilepsy, structural brain disorders, psychiatric and cardiac disease, and disability, were all associated with increased total costs according to the univariate analyses, which are detailed in Table 6. Younger age, polytherapy with two or more ASDs, TSC manifestations, such as epilepsy, psychiatric disease, and disability, were associated with higher indirect costs, and older age, polytherapy with two or more ASDs, TSC manifestations such, as epilepsy, psychiatric and kidney disease, and disability, were associated with increased nursing care-level costs according to the univariate analyses.

**Table 6:** Univariate and multivariate analysis of cost-driving factors for total direct, total indirect and nursing care level costs (3-months period in 2019 Euro)

	n	Total direct costs in €	Median	SD	*p* value^§^	Total indirect costs in €	Median	SD	*p* value^§^	Nursing care level costs in €	Median	SD	*p* value^§^
Gender					0.207				0.839				0.809
Male	95	5213	2655	5927		3291	1371	4063		1149	948	1002	
Female	89	4667	1873	6259		3071	548	4612		1178	1635	1032	
Age					0.632*				0.099*				0.005*
0 to 3 years	24	4554	2786	5358		4396	3231	4771		589	474	645	
4 to 10 years	78	5047	2519	5970		2923	269	4635		1127	948	1027	
11 to < 22 years	73	5088	1556	6578		2796	645	3687		1358	1635	1042	
Number of antiseizure drugs					< 0.001^#^				0.002				< 0.001
≥ 2	94	6829	3205	7147		4172	2553	4814		1458	1635	956	
0–1	90	2985	1256	3871		2153	215	3488		855	0	985	
mTOR inhibitors intake					< 0.001^#^				0.087				0.097
Yes	49	11,459	11,358	5945		2322	430	3795		1367	1635	1023	
No	135	2586	1346	4085		3497	1371	4476		1089	948	1004	
Seizures					< 0.001				0.006				0.002
Recurrent seizures	88	6852	3765	6821		4014	2258	4768		1408	1635	992	
Seizure free > 1 year or no seizures	96	3204	1196	4707		2423	215	3744		938	948	984	
Epilepsy					0.002				0.043				< 0.001
Yes (91.8%)	169	5230	2384	6213		3351	860	4390		1251	1635	1003	
No (8.2%)	15	1778	711	2839		1308	0	3051		172	0	473	
Structural brain disorders					0.022				0.059				0.170
Yes (83.2%)	153	5321	2388	6293		3369	860	4424		1211	1635	993	
No (16.8%)	31	3111	1211	4541		2273	0	3742		925	0	1096	
Psychiatric disorders					< 0.001				< 0.001^#^				< 0.001^#^
Yes (51.1%)	94	6372	2802	6564		4387	2848	4883		1727	2184	890	
No (48.9%)	90	3463	1213	5157		1929	161	3233		574	0	774	
Heart and circulatory manifestations					0.008				0.133				0.059
Yes (66.8%)	123	5755	2655	6678		3550	1097	4394		1257	1635	1044	
No (33.2%)	61	3324	1611	4245		2447	430	4125		973	948	930	
Kidney and urinary tract manifestations					0.392				0.113				0.010
Yes (53.3%)	98	4714	1993	6227		3552	1075	4380		1341	1635	1028	
No (46.7%)	86	5217	2389	5931		2765	269	4252		960	948	964	
Skin manifestations					0.150				0.659				0.110
Yes (85.9%)	158	4676	1890	6106		3139	645	4206		1212	1635	1018	
No (14.1%)	26	6606	4946	5749		3457	323	5082		865	474	950	
Other disorders					0.600				0.899				0.991
Yes (29.9%)	55	4134	2000	4685		2868	548	3808		1166	948	982	
No (70.1%)	129	5296	2122	6570		3319	645	4537		1162	1635	1031	
Level of disability					< 0.001				< 0.001^#^				< 0.001^#^
None or ≤ 60%	61	3515	1057	5560		1544	0	3235		187	0	529	
70–100%	123	5660	2495	6221		3998	2742	4574		1647	1635	831	
Total Disorders					0.009*				0.016*				< 0.001*
1–2 Manifestations (8.7%)	16	1282	894	1365		1593	0	3805		323	0	604	
3 Manifestations (13%)	24	3705	1522	4194		1637	0	2811		403	0	736	
4 Manifestations (17.9%)	33	5916	2085	6097		3475	1468	5171		1139	948	900	
5 Manifestations (32.6%)	60	5839	2432	7021		3087	860	4037		1367	1635	972	
6–7 Manifestations (27.8%)	51	5011	2403	6168		4337	2957	4561		1560	1635	1027	

^§^Mann–Whitney-U-test

*Kruskal–Wallis-test; SD = standard deviation

^#^Significant predictor in multivariate analysis after Bonferroni correction

Overall, total direct, total indirect, and nursing costs increased as the number of affected organ systems increased (Table 6).

Multiple regression analyses revealed that polytherapy with two or more ASDs and the use of mTOR inhibitors were independent cost-driving factors for total direct costs. After applying a Bonferroni correction for eight comparisons, the significance threshold for the p-value was set to 0.00625, and the variables were able to explain 53% (R^2^) of the total variance. Younger age and psychiatric disease were independent cost-driving factors for total indirect costs (corrected *p* < 0.00625; R^2^ = 19%). Psychiatric disease and disability were independent cost-driving factors for nursing care-level costs (corrected *p* < 0.007; R^2^ = 57%).

## Discussion

This detailed, multicenter, COI study is based on a large sample of 184 patients and their caregivers within a single, national healthcare system and contributes important new information regarding the costs and cost-driving factors associated with TSC in Europe. To enable comparisons with other COI studies, we aimed to capture the most comprehensive set of cost items related to epilepsy and other TSC organ manifestations [3, 34].

Previous studies have reported direct cost estimates for patients with TSC in Europe [11, 13, 35, 36] and North America [32, 33, 37–40]; however, no previous studies have provided indirect cost estimates for caregivers whose working lives are affected by TSC in their children [3]. The wider societal impacts determined for the indirect costs incurred by caregivers of patients with TSC were substantial, calculated at an annual mean of EUR 12,700, which exceeds the indirect costs of EUR 5250 reported for the parents of a general German population of children with epilepsy [21] but compares well with the EUR 19,150 calculated in Germany for the caregivers of children and adolescents with Dravet syndrome, a severe early-onset epileptic encephalopathy [41]. The results of the current study were comparable with the outcomes reported by other German studies examining refractory epilepsy [21, 41], which may be due to the use of the same methodology. The high indirect costs suggest that persisting seizures refractory to ASD polytherapy, psychiatric disease, and disability reflect a cost driver among patients with epileptic encephalopathies [42–44]. In line with these findings, we were able to show that TSC manifestations, such as epilepsy, psychiatric disease, and disability, were associated with increased indirect costs, indicating a high strain on the working lives of caregivers. We calculated the indirect costs for adolescents to be EUR 1002 per three months; however, this finding should be viewed with caution due to the limited number of adolescents of working age in our study population.

Another particular contribution of this study was the collection of data regarding the nursing requirements of the study population, as measured by the care grade allowances, which were among the most important cost components assessed, associated with annual expenditures totaling EUR 4650. This finding reinforced the significant effects of different organ manifestations, together with seizure-related costs, which were also reported by Skalicky et al. [40].

Generally, the results of this study indicated that the management of TSC results in considerable resource use, exceeding the costs of German epilepsy patients, in general. Epilepsy was the major TSC organ manifestation, reported in 91.8% of our cohort. Total annual direct healthcare costs were estimated at EUR 19,800 in this study, excluding care grade allowances. The direct costs related to TSC were primarily the result of mTOR inhibitor use (46.6% of total direct costs, EUR 2309 per three months) and hospitalization (20.8% of total direct costs, EUR 1027 per three months) and were impacted far less by ASD use (5.3%, EUR 260) [45]. mTOR inhibitors were used by 49 children and adolescents (26.6% of the cohort); therefore, our study provides unique COI data, reflecting the introduction and wide use of this drug class. The cost of mTOR inhibitor use is likely to decrease in the future as generic formulations become available, a phenomenon that has been well-demonstrated for other ASDs [25, 46]. Although our study appears to agree with other COI studies regarding the contributions of hospitalization and ASD use [11, 13, 32, 33, 35, 36, 39, 40, 47, 48] (for details, please refer to Table 7) performing direct comparisons against studies from different settings and different countries proved to be difficult, as the observed variations were likely associated with a variety of contributing factors including differences in definition, policy, measurements, and population (such as the combination of TSC/epilepsy cohorts). Furthermore, the resource use might change over time according to changes in organ manifestation during the lifetime of TSC patients [3, 49]. Overall, the medical and care-related expenditures among patients with TSC are high and appear to be driven by the severity of each of the multiple disease manifestations (Additioanl file 1). Preventive treatment with new and thus expensive disease-modifying drugs may be outweighed by reductions in the substantial direct and indirect costs, however disease-modifying treatment would have to start in early childhood [10, 50, 51].

**Table 7:** Studies on direct and indirect costs in children and adolescents with TSC

	Grau et al. Current study	Betts et al. 2020 [48]	Chu et al. 2020 [47]	Skalicky et al. 2018 [40]	Song et al. 2017 [32]	Shepherd et al 2017 [11]*	Kingswood et al. 2016 [13]*	Kingswood et al. 2016 [36]*	Wilson et al. 2016 [39]	Sun et al. 2015 [33]	Vekeman et al. [35]
Study design	multicenter, r	multicenter, r	multicenter, r	multicenter, p	multicenter, r	multicenter, r	multicenter, r	multicenter, r	multicenter, r	multicenter, r	monocenter, r
Costing year	2019	2019	2017	2012	2013	2014	2014	2014	n.r	2010	2012
Country (city)	Germany	USA	Hong Kong	USA	USA	United Kingdom	United Kingdom	United Kingdom	USA	USA	Netherlands (Utrecht)
Group	All TSC	TSC and epilepsy	All TSC	All TSC	TSC and AML	TSC and epilepsy	All TSC	TSC and kidneys	All TSC	TSC and SEGA surgery	TSC and kidneys
Number of patients	184	2028	284	179	256	209	286	79	5655	47	369
Study population	C	C & A	C & A	C	C	C & A	C & A	C & A	C & A	C & A	C & A
Patients with epilepsy	91.8%	100%	71.3%	n.r	n.r	100%	n.r	n. r	41.2%	91%	n.r
Age in years (median)	0.7–21.8 (9.8)	Mean 25.3	0.45–89.9 (27.2)	0–18 (6.0)	Mean 9.7, 6.9	Mean 26.8	Mean 31.5	Mean 38.3	Mean 22.3	Mean 11.6	Mean 42.8^7^
Patients with ASDs	83.7%	89.5%	n. r	n.r	n.r	88%	42.7%	68.4%	n.r	n.r	n.r
Patients with mTOR-inhibitors	26.6%	10%	16.5%	n.r	n.r	n.r	n.r	n.r	n.r	n.r	n.r

	Mean PPPY	Mean PPPY	Mean PPPY	Median PPPY	Mean PPPY	Mean PPPY	Mean PPPY	Mean PPPY	Median	Mean PPPY	Mean PPPY
Total direct costs	EUR 19,796	n.r	n.r	n.r	USD 35,381–29,240^4^	GBP 4778^5^	GBP 4227^5^	GBP 5054^5^	n.r	USD 8543–85,397^6^	EUR 1275–31,916^8^
Medication	EUR 10,632	USD 18,836	n.r	USD 1800^2,3^	USD 7445–11,002^4^	*no specific amount*	GBP 595^5^ *(only primary care)*	GBP 869^5^ *(only primary care)*	n.r	USD 1300–2338^6^	EUR 429–1508^8^
* ASDs*	EUR 1040	USD 12,866	n.r	n.r	n.r	n.r	n.r	n.r	n.r	n.r	n.r
* mTOR inhibitors*	EUR 9236	USD 4028	n.r	n.r	n.r	n.r	n.r	n.r	n.r	n.r	n.r
Hospitalization	EUR 4108	USD 2106	USD 5819^1^	USD 1675^2, 3^	USD 10,368–8901^4^	*no specific amount*	GBP 2181^5^	GBP 2350^5^	USD 14,807	USD 3770–71,562^6^	n.r
Ancillary therapies	EUR 1628	n.r	n.r	USD 1200^2,3^	n.r	n.r	n.r	n.r	n.r	n.r	n.r
Outpatient treatment	EUR 1384	USD 13,455	USD 1414^1^	USD 400^2^	USD 17,000–9011^4^	*no specific amount*	GBP 645^5^	GBP 690^5^	n.r	USD 3473–11,497^6^	n.r
ER visists	n.r	USD 1535	USD 116^1^	USD 400^2^	USD 568–326^4^	n.r	n.r	n.r	n.r	n.r	n.r

	Mean caregivers PY			Mean caregivers							
Total indirect costs in caregivers	EUR 12,736	n. r	n. r	42%^9^	n. r	n. r	n. r	n. r	n. r	n.r	n.r
Quit work	EUR 6352	n.r	n.r	n.r	n.r	n.r	n.r	n.r	n.r	n.r	n.r
Reduction of working hours	EUR 4780	n.r	n.r	n.r	n.r	n.r	n.r	n.r	n.r	n.r	n.r
Days off due to TSC	EUR 1604	n.r	n.r	n.r	n.r	n.r	n.r	n.r	n.r	n.r	n.r

p = prospective; r = retrospective; C = Children; A = Adults; n.r. = not reported; PPPY = per person/per year; PY = per year; ASD = antiseizure drug

^1^10% of actual expenses, government subsidized more than 90%

^2^"out-of-pocket" direct spending

^3^Calculated

^4^The first amount is from commercial cohort, the second one from medicaid cohort

^5^Calculated for one year, original cost figure given for a 3 year period, excluding GP administartion encounters

^6^From SEGA pre-surgery to post-surgery period

^7^Calculated across all CKD stages

^8^The first amount is from CKD stage 1, the second one from CKD stage 5, overall mean PPPY costs for AML: EUR 1451-3243

^9^Proportion of parents with time missed or greater productivity loss

*Same study cohort

### Limitations

Potential limitations associated with the questionnaire used in this study include recall bias regarding the three-month-old events, which might result in incomplete and underestimated costs. However, a validation of three-months recall for all items and of twelve-months recall regarding high-impact events like hospitalization or emergency calls with a prospective diary covering three months showed an excellent overlap in patients with Dravet syndrome, another developmental and epileptic encephalopathy [41]. Furthermore, although the sample consisted of patients recruited from multiple clinics and centers across Germany and through the patient advocacy group, whether the sample is representative of TSC patients in Germany remains challenging to determine due to the difficulty of estimating patient numbers for rare diseases. As the patients were approached by different physicians such as neuropediatricians, neurologists and nephrologists as well as through the patient advocacy group, we cannot reliably calculate a response rate as some patients were contacted several times. The use of costly treatments, such as everolimus, might be overestimated because several university centers participated in this study. In addition, the interpretation of the cost driver analysis must consider the limited sample size; however, the significance of multiple organ manifestations in the current study aligns well with earlier studies, which reported that the number of organ manifestations was a significant cost driver [11]. In addition, skewness was observed in the cost calculations, indicated by the disparities observed for some mean and median costs. We did not conduct any sensitivity analyses to test for uncertainty or any alternative costs. The major strength of the present study was the sample size of 184 patients and caregivers, which can be considered a large sample, given the relative rarity of TSC, as well the inclusion of the relatively new class of mTOR inhibitors in the cost analysis for the treatment of children and adolescents with TSC.

## Conclusions

Medical and care-related expenditures among patients with TSC are high and appear to be driven by the severity of disease manifestations. In the long term, high direct costs might be outweighed by the preventive potential and multi-organ benefits of newer therapies. Productivity losses represent a major source of costs and may be addressed by improving socio-medical support systems and therapeutic interventions. Efforts should be focused on reducing work absenteeism and the rate at which caregivers stop working entirely while maintaining the quality of care provided to children and adolescents with TSC.

## Supplementary Information

**Additional file 1.** **Supplementary Table 1.** Direct costs related to TSC manifestations.

## Data Availability

The datasets analysed during the current study are available from the corresponding author on reasonable request.
