# Supplementary material for: Direct and indirect costs and cost-driving factors of Tuberous sclerosis complex in children, adolescents, and caregivers: a multicenter cohort study
Source: Orphanet J Rare Dis. 2021 Jun 21;16:282. doi: 10.1186/s13023-021-01899-x (PMC8218507; doi:10.1186/s13023-021-01899-x)
Supplement: Supplementary file 1 — Additional file 1. Supplementary Table 1. Direct costs related to TSC manifestations. [file 13023_2021_1899_MOESM1_ESM.docx]

Appendix Tables

Supplementary Table 1 Direct costs related to TSC manifestations

| Costs in organ systems | Grau et al. current study | Kingswood et al. 2016 a* | Kingswood et al. 2016 b* | Shepherd et al 2017* |
| --- | --- | --- | --- | --- |
|  | Mean PPPY | Mean PPPY | Mean PPPY | Mean PPPY |
| Epilepsie / nervous system^1^ | EUR 20,920 | GBP 4918 ^1^ | GBP 5844 ^1^ | GBP 1684 ^1, 2^ |
| Structural brain disorders^1^ | EUR 21,284 | GBP 7603 ^1^ | n.r. | GBP 8045 ^1^ |
| Psychiatric disorders^1^ | EUR 25,488 | GBP 5325 ^1^ | n.r. | n.r. |
| Heart and circulatory manifestations^1^ | EUR 23,020 | GBP 5527 ^1^ | n.r. | GBP 6124 ^1^ |
| Kidney and urinary tract manifestations^1^ | EUR 18,856 | GBP 5232 ^1^ | GBP 1200 ^1, 2^ | n.r. |
| Skin manifestations^1^ | EUR 18,704 | GBP 4417 ^1^ | GBP 5844 ^1^ | GBP 4644 ^1^ |
| Respiratory manifestations^1^ | EUR 0 | GBP 13,664 ^1^ | n.r. | GBP 13,110 ^1^ |

^1^Calculated for one year, original cost figure given for a 3 year period, including GP administration encounters

^2^Only for one manifestation, baseline costs, they are added to all other manifestations

*Same study cohort
